# HPV-18-Immortalised Cells Require the Downregulation of the SncmtRNA-2/Hsa-miR-620 Axis During Cell Transformation

**DOI:** 10.3390/medicina62010110

**Published:** 2026-01-04

**Authors:** Emanuel Jeldes, Manuel Varas-Godoy, Paulina González-Chacón, América V. Campos, Alberto J. M. Martín, Camilo Villaman, Ángel Roco-Videla, Jaime Villegas Olavarría, Claudio Villota Arcos

**Affiliations:** 1 Centro Científico y Tecnológico de Excelencia Ciencia y Vida, Santiago 8340148, Chile; ejeldes@ed.ac.uk; 2 Centre for Inflammation Research, Institute for Regeneration and Repair, University of Edinburgh, Edinburgh EH166 4UU, UK; 3 Centro de Biología Celular y Biomedicina (CEBICEM), Facultad de Ciencias, Universidad San Sebastián, Santiago 8420524, Chile; manuel.varas@uss.cl; 4 Centro Ciencia & Vida, Fundación Ciencia & Vida, Santiago 8320000, Chile; 5 Escuela de bioquímica, Facultad de Ciencias de la Vida, Universidad Andrés Bello, Santiago 8370000, Chile; paulina.gonzalezchacon@gmail.com; 6 Cancer Research UK Scotland Institute, Glasgow G61 1BD, UK; a.campos-gonzalez@crukscotlandinstitute.ac.uk; 7 Escuela de Ingeniería, Facultad de Ingeniera, Arquitectura y Diseño, Universidad San Sebastián, Santiago 8420524, Chile; alberto.martin@uss.cl; 8 Centers of Research Excellence in Science and Technology, Science & Life, Santiago 8580702, Chile; c.villaman@gmail.com; 9 Facultad de Ingeniería, Universidad Católica de la Santísima Concepción, Concepción 4090541, Chile; aroco@ucsc.cl; 10 Dirección de Desarrollo y Postgrados, Universidad Autónoma de Chile, Galvarino Gallardo 1983, Santiago 7500138, Chile; 11 Escuela de Medicina Veterinaria, Faculty de Ciencias de la Vida, Universidad Andrés Bello, Santiago 8370000, Chile; jaime.villegas@unab.cl; 12 Escuela de Nutrición y Dietética, Facultad de Ciencias de la Salud, Universidad Bernardo O’Higgins, Santiago 8370993, Chile; 13 Escuela de Medicina, Facultad de Ciencias Médicas, Universidad Bernardo O’Higgins, Santiago 8370993, Chile

**Keywords:** HPV, hsa-miR-620, Ras, cell transformation, SncmtRNA-2

## Abstract

*Background and Objectives*: Non-coding RNAs (ncRNAs) are genetic transcripts that do not produce proteins but are increasingly recognised for their roles in cellular processes and disease. Specifically, ncRNAs are implicated in the landscape activation of molecular triggers for different diseases, including cancer and viral infections. The function of Sense non-coding mitochondrial RNA-2 (SncmtRNA-2) is currently unknown. This study aims to investigate the roles of SncmtRNA-2 and hsa-miR-620 in Ras-induced cellular transformation. *Materials and Methods*: The study utilized isoforms V, K, and H of the Ras oncogene and analysed the expression of SncmtRNA-2 and hsa-miR-620 in response to Ras activity. Additionally, both in silico and in vitro analyses were performed to assess whether PML mRNA is a putative target of hsa-miR-620 although direct binding to the PML 3′UTR was not experimentally tested. *Results*: The research demonstrated that transformation induced by Ras isoforms V, K, and H resulted in decreased expression of both SncmtRNA-2 and hsa-miR-620. Further investigation revealed that hsa-miR-620 is produced by the processing of SncmtRNA-2. It was also shown that Ras increases the expression of Promyelocytic Leukemia Protein (PML). In silico prediction combined with miR-620 gain and loss of function experiments supports PML as a putative hsa-miR-620 target. *Conclusions*: Ras promotes cellular transformation by decreasing the expression of SncmtRNA-2 and hsa-miR-620, which may contribute to increased PML expression, suggesting but not demonstrating a possible regulatory relationship among these molecules in HPV-immortalised cells. These results highlight a potential SncmtRNA-2/miR-620/PML axis that requires further validation through direct interaction assays and functional necessity/sufficiency experiments.

## 1. Introduction

Human papillomavirus (HPV) infection, particularly involving the high-risk HPV-16 and HPV-18 variants, is a major cause of cervical cancer, which ranks among the leading causes of cancer-related deaths in women worldwide [1,2]. The process of HPV-induced malignant transformation is mainly driven by the viral oncoproteins E6 and E7. These oncoproteins disrupt the functions of tumour suppressors p53 and pRb, leading to in cell cycle dysregulation, genomic instability, and resistance to apoptosis [1,3]. This process is further supported by the integration of the viral genome into the host cell DNA, which promotes persistent oncoprotein expression and increases the likelihood of progression to invasive carcinoma [2]. In addition to the classical mechanisms mediated by viral proteins, mitochondrial non-coding RNAs (ncmtRNAs) also play a key role in the HPV-associated cellular transformation process. These include sense (SncmtRNA) and antisense (ASncmtRNA-1 and -2) transcripts, which exhibit significant differences in expression between normal and cancerous cells. While SncmtRNA-1 (S1) is present in both normal and proliferating tumour cells, ASncmtRNAs are markedly suppressed in tumour cells, suggesting a regulatory role in tumour progression [4,5]. Studies have shown that silencing ASncmtRNAs in human tumour cells induces significant cell death by triggering apoptosis, along with decreased levels of key cell cycle regulators, including CDK1, CDK4, Cyclin B1, and Survivin, confirming their role in controlling cell proliferation and survival [5]. The repression of ASncmtRNA-2 (AS2) during HPV-mediated cellular immortalisation has been linked to the expression of SncmtRNA-2 (S2), a newly identified sense transcript in keratinocytes infected with HPV-16 and HPV-18. This shift in mitochondrial expression occurs early in cellular transformation, implying a necessary role for silencing ASncmtRNAs to maintain a dysregulated proliferative state [4].

Previously, we reported that HPV E2 represses ASncmtRNAs in human HPV-18-immortalised cells. This effect can be reversed by inhibiting E2 expression, suggesting that E2 is essential for maintaining repression of this mitochondrial pathway during immortalisation [6]. The SncmtRNA-2/hsa-miR-620 axis has also been identified as a regulatory pathway in cervical cancer, in which overexpression of SncmtRNA-2 represses ASncmtRNAs and increases the expression of the human oncogenic microRNA hsa-miR-620 [5,7].

Otherwise, hsa-miR-620 has been implicated in various tumour processes, including cell proliferation and migration. In cervical cancer, hsa-miR-620 has been shown to promote malignant characteristics by repressing suppressor genes, and its expression can be regulated by circular RNAs such as circSMARCA5, which acts as a sponge for this microRNA [8]. This interaction reveals a complex post-transcriptional regulatory network, in which SncmtRNA-2 may act as a central node, deregulating hsa-miR-620 and repressing ASncmtRNAs, thereby creating a molecular environment favorable to HPV-induced cellular transformation [7]. The SncmtRNA-2/hsa-miR-620 axis, therefore, plays a fundamental role in cellular transformation and can be analysed from the perspective of RAS-mediated oncogenic signalling, a pathway frequently activated in various types of cancer. RAS genes, particularly KRAS, are mutated in up to 20% of human cancers, and their activation stimulates multiple effector pathways, including the RAF-MEK-ERK and PI3K-AKT-mTOR pathways, which regulate cell growth, differentiation, and survival [9,10]. The importance of RAS in cancer development depends on its relative abundance, subcellular localisation, and activation dynamics, which can be indirectly modulated by the mitochondrial environment and the expression of non-coding RNA (ncRNAs), such as SncmtRNA-2 [6,11,12]. Since RAS signalling can either induce or inhibit cellular senescence depending on the context, the deregulation of mitochondrial ncmtRNAs could act as a critical modulator in this balance. The expression of SncmtRNA-1 has been linked to active cell proliferation, whereas the expression of ASncmtRNAs seems to inhibit cell cycle progression and promote senescence and apoptosis events [5]. In this context, HPV suppression of ASncmtRNA-2 would prevent senescence and enable a sustained proliferative state, thereby encouraging neoplastic progression.

The aim of this research is to determine the role of mitochondrial non-coding transcripts (SncmtRNA-2) and the human microRNA miR-620 (hsa-miR-620) in the cellular transformation of cells immortalized by human papillomavirus (HPV), particularly through inducing this transformation via overexpression of RAS family oncogenes.

## 2. Materials and Methods

### 2.1. Cell Culture

Human foreskin keratinocytes (Lonza, Basel, Switzerland) were cultured in Keratinocyte Serum-free Medium (KSFM; Invitrogen, Waltham, MA, USA). HPV-derived tumour cell lines, HeLa (ATCC-CCL-2) and SiHa (ATCC-HTB-35), were cultured in DMEM supplemented with 10% FBS. HPV-immortalised cells, HFK698 and 18Nco, were cultured in 3:1 media (KSFM:10% FBS DMEM). HPV-16-immortalised cells (HFK698) and HPV-18-immortalised cells (18Nco and HF18) were kindly donated by Dr. Luisa L Villa (Ludwig Institute for Cancer Research, São Paulo, Brazil). All cell lines were cultured at 37 °C in a 5% CO_2_ atmosphere until they reached 80–90% confluence.

### 2.2. Determination of the ncmtRNAs Expression by RT-PCR

Total RNA was extracted from cells using TriZol (Invitrogen) according to the manufacturer’s instructions. Five micrograms of RNA were treated with 2 U of TURBO DNase RNase-free (Ambion, Austin, TX, USA) for 30 min at 37 °C. Reverse transcription was performed using 100 ng of freshly prepared RNA, 50 ng of random hexamers, 0.5 mM dNTPs, and 200 U of reverse transcriptase (M-MLV, Invitrogen) in a final volume of 20 µL. Then, 2 µL of the cDNA were amplified via PCR in a mixture containing 2.5 U of GoTaq (Promega, Madison, WI, USA), 1.5 mM CaCl_2_, 0.4 mM dNTPs, 1 µM of forward primer, and 1 µM of reverse primer in a final volume of 50 µL. The PCR programme comprised the following steps: 100 °C for 10 min, 70 °C for 10 min, 80 °C for 10 min, and 94 °C for 5 min. This was followed by 30 cycles at 94 °C for 1 min, 58 °C for 1 min, and 72 °C for 1 min. The amplicons were then analyzed by electrophoresis on a 2% agarose gel. The primers used to amplify the ncmtRNAs were designed based on the sequence of human mitochondrial 16 S rRNA (GenBank Accession Number V00662): for S1; fw: 5′-AGGTTTAGCCAAACCATT-3′ and rev: 5′-AAGGTGGAGTGGGTTTGGGGC-3′. For S2; fw: 5′-AGGTTTAGCACCGCAAGGG-3′ and rev 5′-AAGGTGGAGTGGGTTTGGGGC-3′. For AS2; fw: 5′-ACCGTGCAAAGGTAGCATAATCA-3′ and rev: 5′-CAAGAACAGGGTTTGTTAGG-3′. For AS1; fw: 5′-TAGGGATAACAGCGCAATCCTATT-3′ and rev: 5′-CACACCCACCCAAGAACAGGGAGGA-3′. The expression control, 18 S rRNA; fw: 5′-GTAACCCGTTGAACCCCATT-3′ and rev: 5′-CATCCAATCGGTAGTAGCG-3′.

### 2.3. Overexpression of Ras in HPV Immortalised Keratinocytes by Lentiviral Vectors

The oncogenic sequences of H, K, or V-Ras were synthesised and cloned into the lentiviral vector pLVX-IRES-ZsGreen1 (GenScript, Piscataway, NJ, USA). Lentiviral particles were produced by co-transfecting the lentiviral vectors encoding the different isoforms of RAS, or an empty vector pLVX-IRES-ZsGreen1 (as a control), with packaging plasmids in HEK-293T cells under standard conditions [9]. 18Nco and HFK698 cells were transduced with the lentiviral particles using a multiplicity of infection (MOI) of 5. After 24 h, the high population of ZsGreen-expressing cells was selected by cell sorting (BD FACS Aria II cell sorter), expanded, and used to seed monolayer cultures.

### 2.4. Detection of Ras Protein by Western Blot

The pellet of cells was resuspended directly in 100 µL of p300 buffer (containing 20 mM NaH2PO4, 250 mM NaCl, 30 mM NaPPi, 0.1% Nonidet P-40, 5 mM EDTA, and 5 mM dithiothreitol) for 30 min at 4 °C, followed by centrifugation. Total protein was quantified using the BCA protein assay reagent (ThermoFisher Scientific, Waltham, MA, USA) according to the manufacturer’s instructions. Thirty micrograms of total protein were then denatured in Laemmli sample buffer, boiled, and separated by 12% SDS-PAGE. Subsequently, transfer to nitrocellulose membranes was performed overnight. Membranes were incubated in blocking solution (TBS-Tween with 10% milk) for 1 h at room temperature, then incubated overnight with primary antibodies: anti-RAS 1:1000 (Abcam, Boston, MA, USA), anti-β-Actin 1:1000 (Abcam), anti-PML 1:250 (Santa Cruz, Dallas, TX, USA). Afterwards, they were washed with TBS-Tween. Incubation with secondary antibodies coupled with alkaline phosphatase (1:5000, Abcam) was performed for 1 h, followed by washing in TBS-Tween. Membranes were revealed using the EZ-ECL system (Biological Industries, Cromwell, CT, USA) on a C-DiGit Blot Scanner (LI-COR Biosciences, Lincoln, NE, USA).

### 2.5. Determination of DNA Synthesis

To estimate cell proliferation rates in cells overexpressing RAS proteins compared to those with an empty vector (control), we used the Click-iT EdU assay (Invitrogen) according to the manufacturer’s instructions.

### 2.6. Transwell Assay (Migration Capacity)

To assess migration capacity using a matrix barrier, cells were detached with trypsin, washed, and resuspended at a density of 5 × 10^5^ cells/mL in DMEM containing 0.1% FBS. One hundred microliters of the cell suspension were then plated onto 6.5 mm-diameter (8 µm pore size) polycarbonate membrane tissue culture inserts (Corning, Tewksbury, MA, USA). DMEM supplemented with 5% FBS was added to the lower chamber to prevent chemical bias during migration, and cells were allowed to migrate for 4 h. Cells were stained with crystal violet. Cells on the lower side were imaged and quantified for each of the 3 independent experiments. ImageJ 1.54r (NIH) was used to count stained nuclei above background and with a size greater than 50 pixels.

### 2.7. Colony Formation in Soft Agar

One characteristic property of tumour cells is their ability to grow independently of anchorage. To determine whether overexpression of RAS induced cell transformation in HPV-immortalised cells, 23 mm Petri dishes with 5 mL of Agar-Nobel-KSFM/DMEM 0.8% were prepared and allowed to solidify overnight at 4 °C. Twenty-four hours later, 104 cells were mixed with 1.5 mL of Agar-Nobel-KSFM/DMEM 0,4%, and the mixture was seeded onto the 23 mm plates previously prepared and cultured at 37 °C in 5% CO_2_. The cells were cultured for twenty-one days. After this, colonies with a size over 100 μm were quantified.

### 2.8. Determination of Hsa-miR-620 Expression by Real-Time RT-PCR

For miRNA expression, total miRNA was extracted using a Purelink miRNA kit (Invitrogen) according to the manufacturer’s instructions. cDNA was synthesised using the NCode^TM^ VILO^TM^ miRNA cDNA Synthesis Kit (Invitrogen) according to the manufacturer’s instructions. The mature hsa-miR-620 expression levels were quantified by real-time PCR using Express SYBR^®^ GreenERTM qPCR SuperMix Universal (Invitrogen) on the Stratagene Mx3000PTM Real-time PCR System (Agilent Technologies, Santa Clara, CA, USA). miRNA expression levels were calculated using the comparative Ct method via MxPro software (Version 4 10), and the data were analysed by ANOVA (GraphPad, version 10.6.1). The relative expression levels were normalised to U6 and hsa-miR-21. The primer sequences: U6 fw: 5′-TGCGGGTGCTCGCTTCGGCAGC-3′; hsa-miR-21 fw: 5′-TAGCTTATCAGACTGATGTTGA-3′; hsa-miR-620 fw: 5′-ATGGAGATAGATATAGAAAT-3′.

### 2.9. Silencing of SncmtRNA-1 and 2, PML, and Hsa-miR-620

Antisense oligonucleotides (ASOs) used in this study were synthesised by IDT (Promega, Madison, WI, USA) with 100% phosphorothioate (PS) linkages. The sequences of the ASOs utilized were: Directed against S1 5′-CACCCACCCAAGAACAGG-3′; S2 5′-GTCCTAAACTACCAAACC-3′; PML 5′-TACCTAAAAAATCCCAAACA-3′. Human miR-620 (hsa-miR-620) was silenced using antagomiR-620 (LifeScience, Darmstadt, Germany). Some ASOs were labelled at the 5′ end with Alexa Fluor 488 to assess the transfection efficiency. For ASO treatments, cells were seeded into 12-well plates (Nunc) at 50,000 cells per well and transfected the following day with ASOs at concentrations ranging from 200 to 400 nM using Lipofectamine 2000 (Invitrogen) according to the manufacturer’s instructions. Transfection was allowed to proceed for 48 h under normal culture conditions. PML expression was evaluated by Western Blot. Transfected cells were harvested, washed in ice-cold PBS, and sedimented at 1000× *g* for 10 min at 4 °C. Pellets were suspended in RIPA buffer (10 mM Tris-HCl, pH 7.4, 1% sodium deoxycholate, 1% Triton X-100, 0.1% sodium dodecyl sulfate, ThermoFisher Scientific), containing 1 mM PMSF and 1X protease inhibitor cocktail (Promega), and sonicated before SDS-PAGE immunoblotting. Protein concentration was quantified with the Bradford microplate system Gen5TM EPOCH (BioTek, Winooski, VT, USA) [13]. Thirty micrograms of protein were resolved by SDS-PAGE and transferred onto nitrocellulose membranes overnight at 4 °C. Membranes were incubated with antibodies against β-tubulin (anti-rabbit; 1:1000, Abcam) and PML [H-238] (anti-rabbit; 1:200, Santa Cruz) in 5% BSA overnight at 4 °C. The membranes were washed, and primary antibodies were detected using peroxidase-labelled anti-mouse or anti-rabbit IgG (1:5000, Rockland, Limerick, PA, USA) in 5% BSA for 2 h at room temperature (RT). The blots were visualised with the EZ-ECL system (Biological Industries) on a C-DiGit Blot Scanner (LI-COR Biosciences, Lincoln, NE, USA). The intensity of each band was quantified using ImageJ 1.54r software (NIH).

### 2.10. R6G Treatment

To block mitochondrial metabolic activity, cells were cultured in normal medium supplemented with Rhodamine 6-G (10 µM) for 72 h. The mitochondrial transcription was assessed by RT-PCR amplification of nuclear and mitochondrial mRNAs.

### 2.11. EtBr Treatment

To inhibit mitochondrial DNA transcription, cells were cultured in a normal medium supplemented with Ethidium Bromide (5 µg/mL), Pyruvate (1 nM), and Uridine (50 µg/mL) for 21 days. Mitochondrial transcription was assessed by RT-PCR amplification of nuclear and mitochondrial mRNAs.

### 2.12. Detection of Cytokeratin 14 and 17

Cells were cultured for 16 h in 8-well chamber slides, then washed with PBS and fixed in 4% paraformaldehyde in PBS for 10 min at room temperature. The slides were washed three times with PBS for 5 min each and then incubated with 0.3% Triton X-100 for 10 min at room temperature. Cells were washed three times with PBS for 5 min each, then incubated with 2% BSA in PBS for 30 min to block nonspecific antibody binding. Cells were incubated with primary antibodies (CK14 1:300 and CK17 1:200; Abcam) in 2% BSA in PBS-T in a humidified chamber at room temperature for 1 h. The slides were washed three times with PBS for 5 min each, then incubated with secondary antibodies (Anti-mouse CK14 1:250 and Anti-rabbit CK17 1:200; Abcam) in 2% BSA for 1 h at room temperature in the dark. The slides were washed twice with PBS for 5 min and then incubated with DAPI (1:1000) in PBS for 10 min. Finally, the slides were washed in PBS, mounted in Dako Fluorescent mounting medium (DAKO, Santa Clara, CA, USA), and analysed and photographed using Q-capturePro software (Version 7.4.4.0) in an Olympus BX-51 microscope.

### 2.13. Target Prediction

Firstly, we aligned the SncmtRNA-2 sequence with the hsa-miR-620 sequence to identify similarities between both using SSEARCH2SEQ from Ensembl using default [13]. To identify downstream effectors of hsa-miR-620, we employed TargetScan, which predicts biological targets of miRNAs by searching for conserved 8 mer, 7 mer, and 6 mer sites matching the seed region of each miRNA [14].

### 2.14. Statistical Studies

Experiments were performed at least in triplicate. Results were analysed by two-tailed Student’s t-test and represent the mean ± S.E.M. Significance (*p*-value) was set at the nominal level of *p* < 0.05 or less.

## 3. Results

### 3.1. Characterisation of SncmtRNA-2

**Figure 1:**
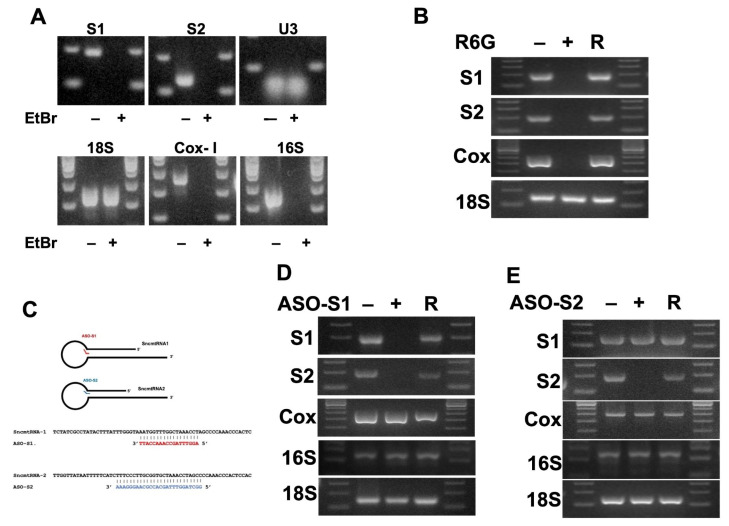
ncmtRNAs expression in HPV-immortalised cells transduced with RAS-coding retroviral vectors. 18Nco cells were incubated with EtBr (**A**) or R6G (**B**) to disrupt mitochondrial transcription. Bioinformatic analysis revealed that the sequence of S2 is 80–90% conserved with the sequence of S1; therefore, we hypothesised that S2 could be a product of S1 processing (**C**). S1 and S2 were amplified by RT-PCR from total RNA of control (−), treatment (+), and recovered (R) samples. U3 and 18 S rRNA were used as controls, whereas 16 S rRNA and Cox mRNA served as mitochondrial transcripts. 18Nco cells were incubated with oligonucleotides to silence S1 (**D**) or S2 (**E**). Sense transcripts were amplified by RT-PCR from total RNA of control (−), ASO-treated (+), and recovered (R) conditions. Cox and 18 S rRNA were used as controls.

The expression of ncmtRNAs is strongly linked to cell proliferation status [11]. Although transformed cells exhibit a marked decrease in ASncmtRNA expression, they retain SncmtRNA-1 (S1) expression [4,11]. We previously reported differential expression of ncmtRNAs in normal keratinocytes, immortalised cells, and HPV transformed cells [6]. The expression levels of sense and antisense ncmtRNAs were determined by RT-PCR, as described previously [6,7]. To investigate the mitochondrial origin of SncmtRNA-2 (S2), HPV-18 immortalised cells (18Nco) were cultured in media supplemented with Rhodamine 6G or Ethidium Bromide, which deplete mitochondria and consequently reduce mitochondrial RNA levels (Figure 1A,B). Although Ethidium Bromide treatment resulted in the loss of 16 S rRNA and Cox-I mRNA expression (mitochondrial RNA, Figure 1A), it did not affect the transcription of nuclear RNAs such as U3 and 18 S rRNA (Figure 1A). Expression of SncmtRNAs (S1 and S2) was also abolished in cells treated with EtBr (Figure 1A). Similar effects were observed with R6G treatment (Figure 1B). Bioinformatic analysis revealed that the sequence of S2 is 80–90% conserved relative to that of S1. Therefore, we hypothesised that S2 may be a processed product of S1 [9]. To test this hypothesis, we silenced S1 (ASO-S1) expression and measured the levels of both S1 and S2 transcripts (Figure 1C). ASO-S1 silencing reduced the expression of both transcripts, S1 and S2 (Figure 1D,E).

### 3.2. Overexpression of Ras Induces Transformation in HPV-Immortalised Cells

**Figure 2:**
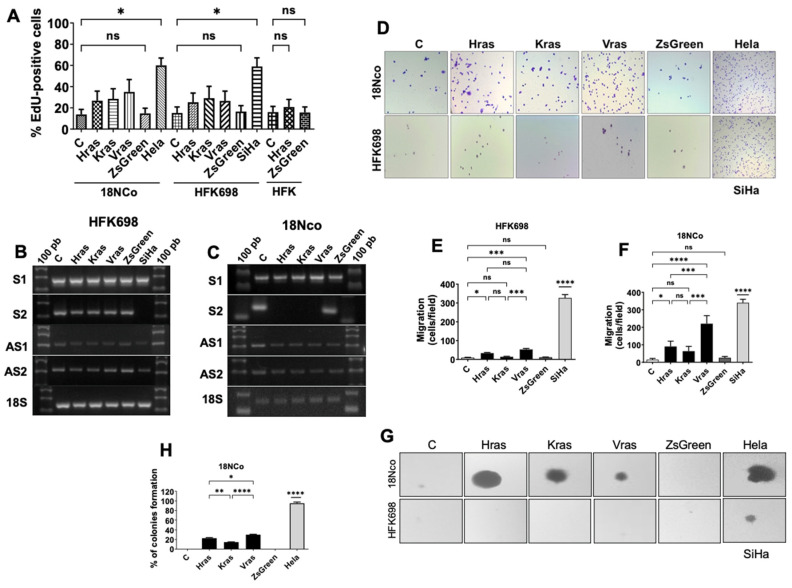
Overexpression of RAS induces cell transformation of HPV-18-immortalised cells and downregulation of SncmtRNA-2. (**A**) DNA synthesis rates were determined by EdU incorporation in HFK698-RAS, 18Nco-RAS, and HFK-HRAS cells. The graph shows the percentage of cells that incorporated Edu. (**B**,**C**) The expression pattern of ncmtRNAs in Ras HFK698 (**D**) or 18Nco (**E**) cells was analysed by RT-PCR. (**D**) The migratory capacity of HFK698-RAS, 18Nco-RAS, and HFK-HRAS cells was assessed using a transwell assay. (**E**,**F**) The graphs show the number of migrating cells per field. (**G**) Transformation of cells overexpressing RAS was evaluated by measuring anchorage-independent growth in HFK698-RAS, 18Nco-RAS, and HFK-HRAS cells. (**H**) The percentages shown in the graph represent colony formation after 21 days, compared to positive controls of tumoural HeLa cells (compared with 18Nco). All experiments were performed in three independent replicates. ns indicates *p* > 0.05; * Indicates *p* < 0.01; ** indicates *p* < 0.001; *** indicates *p* < 0.0001; **** indicates *p* < 0.00001.

Expression of SncmtRNA-2 is associated with the immortalised state and shows a strong decrease in transformed cells. We aimed to induce the transformation of immortalised cells by overexpressing Ras proteins. Overexpression of the oncogenic Ras isoform has been widely reported to induce cell transformation in immortalised cells [15]. To induce transformation of HPV-immortalised cells, HFK698 and 18Nco cells were transduced with a bicistronic lentiviral vector encoding ZsGreen and oncogenic isoforms of H, K, or V-Ras. The transduction efficiency was assessed by measuring ZsGreen expression via flow cytometry. We obtained a range of cell populations, with transduction rates varying from 56.6% to 66.5%, for different Ras isoforms in 18Nco and HFK698 cells. High populations of ZsGreen-positive cells were isolated by cell sorting and maintained in culture (Supplementary Materials Figure S1A). Overexpression of H, K, and V-RAS was determined by Western blot using anti-PAN-Ras antibodies (Supplementary Materials Figure S1B–G). An increase of up to 5-fold of RAS levels in transduced HFK698, 18Nco (Supplementary Materials Figure S1B–E). As a control, normal HFK cells were transduced with the H-Ras coding vector, achieving similar percentages of transduction as HFK698 (Supplementary Materials Figure S1F,G). To determine whether Ras overexpression promotes cell transformation in HPV-immortalised cells, we assessed their DNA synthesis rates, migration ability, and anchorage-independent growth. The overexpression of H-RAS or empty vector (IRES) in HFK did not lead to an increase in Edu incorporation (Figure 2A). However, HFK698 and 18Nco immortalised cells overexpressing Ras showed a higher rate of Edu incorporation compared to non-transduced (C) or empty control transduced (IRES) cells (Figure 2A). The migration ability of HFK698-RAS and 18Nco-RAS was assessed using a Transwell assay (Figure 2D–F). Interestingly, 18Nco-RAS exhibited greater migratory capacity than HFK698-RAS (Figure 2D–F). In both cases, overexpression of RAS resulted in a significant increase in migration compared to non-transduced cells and the empty vector control. To analyse whether RAS-overexpressing cells could induce growth independent of anchoring, we performed a clonogenic soft agar assay (Figure 2G,H). The colonies formed after 21 days of culture were stained and counted. SiHa (transformed by HPV-16) and HeLa (transformed by HPV-18) cells were used as positive controls, while normal HFK cells were used as negative controls. RAS-transduced 18Nco cells (18Nco-RAS) grow at a lower rate than transformed HeLa cells but at a higher rate than Non-transduced 18Nco-RAS cells (C) or empty control (ZsGreen) in soft agar (Figure 2G,H). Surprisingly, although the HFK698 overexpressed RAS protein (HFK698-RAS) at the same levels as 18Nco-RAS (Supplementary Materials Figure S1B–E), it did not grow in soft agar, like HFK698-RAS, non-transduced (C), and empty control (ZsGreen) (Figure 2G). Downregulation of cytokeratin 14 was observed in the tumorigenic transformation of cervical epithelial cells [16]. 18Nco-RAS cells downregulated cytokeratin 14, similar to transformed HeLa cells, but not HFK698, which only showed reduced expression patterns (Supplementary Materials Figure S2). Conversely, in immortalised, RAS-like transformed, and transformed cells, the expression of cytokeratin 17 remained unaffected (Supplementary Materials Figure S2).

### 3.3. Ras-Transformed Cells Downregulate SncmtRNA-2

**Figure 3:**
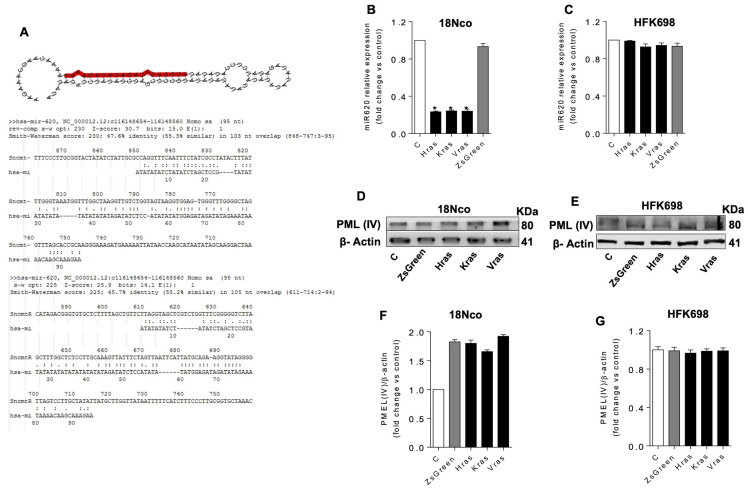
Overexpression of RAS in HPV-immortalised cells. HPV-16/18 immortalised cells were transduced with a lentivirus encoding GFP (ZsGreen) and H, K, and V isoforms of Ras. (**A**) In silico analysis indicates that hsa-miR-620 may be a processing fragment of SncmtRNA-2. The red region highlights the predicted sequence within SncmtRNA-2 that aligns with hsa-miR-620, while the dashed line indicates the putative cleavage/processing site leading to the generation of the miRNA. (**B**,**C**) The expression of hsa-miR-620 was assessed by RT-qPCR in 18Nco and HFK698 cells, both wild-type and expressing Ras isoforms. (**D**,**E**) The expression of PML-IV was assessed by WB in 18Nco and HFK698 cells, both wild-type and expressing Ras isoforms. (**F**,**G**) The graphs display the expression of PML-IV relative to β-actin. The relative expression of PML-IV was calculated from three independent experiments. * Indicates *p* < 0.01 compared toi ZsGreen condition.

To evaluate the expression profiles of ncmtRNAs in transformed and non-transformed cells, total RNA was isolated from HFK698-RAS and 18Nco-RAS, both non-transduced (C) and empty control (ZsGreen) cells (Figure 2B,C). HFK698-RAS maintained the same S2 profile as non-transduced or empty control cells when compared with transformed cell lines, such as SiHa (Figure 2B). However, 18Nco-RAS displayed downregulation of S2 expression to the same level as tumour cell lines, such as SiHa (Figure 2C). In silico analysis demonstrated that the sequences of hsa-miR-620 share 80% nucleotide identity with a segment of the IR sequence of S2 (Figure 3A), suggesting it could be produced through the processing of this sense transcript (Figure 3A). To determine whether the expression of hsa-miR-620 was deregulated during cell transformation, its level of expression was measured using qRT-PCR. HPV-16-immortalised cells (HFK698) control (C), transduced with empty vector (ZsGreen), or over-expressing Ras isoforms exhibited similar levels of hsa-miR-620 (Figure 3C). In contrast, HPV-18-immortalised cells (18Nco) that overexpressed RAS isoforms, but not control cells (C) or those transduced with an empty vector (ZsGreen), exhibited a similar pattern of decreased expression of hsa-miR-620 by approximately fourfold (Figure 3B). These results suggest that the cell progresses from an immortalised to a transformed state. The downregulation of the S2 transcript is required, concomitant with the loss of hsa-miR-620 expression.

### 3.4. PML Is a Putative Target of Hsa-Mir-620

**Figure 4:**
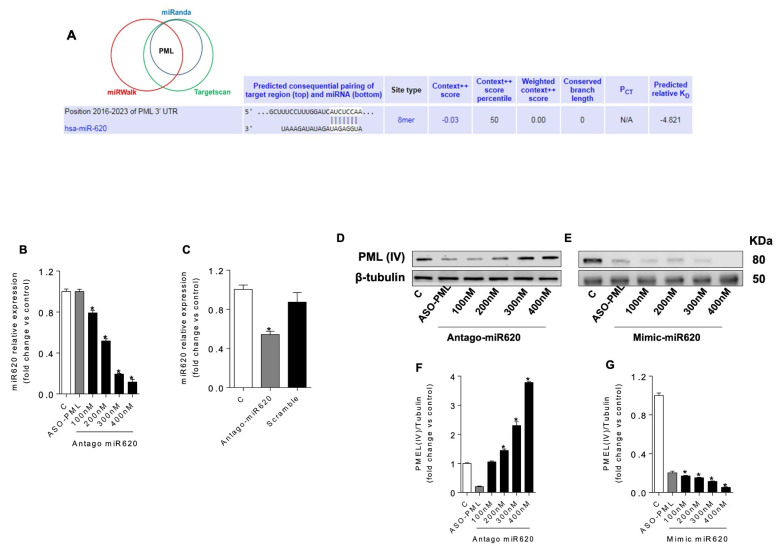
Cell transformation of HPV-18-immortalised cells induces downregulation of hsa-miR-620 and upregulation of PML-IV. (**A**) In silico analysis indicated that the PML is a putative target for hsa-miR-620. (**B**) The expression of hsa-miR-620 was measured by RT-qPCR using total RNA from RAS-18Nco cells treated with antago-miR620. (**C**) The expression of hsa-miR-620 was assessed by RT-qPCR with total RNA from RAS-18Nco-treated cells, using antago-miR620 and a scramble control. (**D**,**E**) The expression of PML-IV was analysed by Western blot using total protein extracts from RAS-18Nco cells treated with ASO-PML and various concentrations of antago-mir620 and mimic-mir620, respectively. (**F**,**G**) The graphs show the expression of PML-IV (from Western blot figures D and E, respectively). Relative expression was determined from three independent experiments. Context++ score and features that contrite to the context ++ score was evaluated as Madeira [13] and Agarwl [14]. * indicates *p* < 0.01.

The Promyelocytic Leukaemia Protein (PML) isoform IV (PML-IV) plays a role in HPV replication and is a target of E6 and E7 oncoproteins [16]. Therefore, we assessed PML-IV expression in HPV-immortalised and Ras-transformed cells. In silico analysis revealed that PML is a putative target of hsa-miR-620 (Figure 4A). To assess this hypothesis, we determined the PML-IV expression by Western blot using total lysates from HPV-immortalised and RAS cell lines (HFK698 and 18Nco). PML-IV expression remained unaffected by RAS overexpression in HPV-16-immortalised cells (HFK698) (Figure 3E,G). However, we observed that PML-IV expression increased almost twofold compared to the control (C) in HPV-18-transformed-like cells overexpressing RAS oncogenic isoforms (18Nco) (Figure 3D,F). There was no significant difference in PML-IV levels between RAS isoforms. To assess whether PML-IV is a putative target of hsa-miR-620, we incubated 18Nco cells (untransduced, mock) with antago-miR620 at increasing concentrations (Figure 4B). Downregulation of hsa-miR-620 was observed starting at 100 nM of Anto-miR620 (Figure 4B). Scramble control treatment did not cause a significant change in hsa-miR-620 expression (Figure 4C). Antisense oligonucleotides targeting PML (ASO-PML) and non-related ones were used as controls (C). PML-IV expression was determined by WB. PML-IV expression increased in correlation with the rising concentration of anto-miR620 (200–400 nM) (Figure 4D,F). As an indirect alternative to validate whether PML-IV is a target of hsa-miR-620, we determined PML-IV expression in cells incubated with increasing concentrations of Mimic-miR620 (Figure 4E,G). PML-IV expression decreased in a concentration-dependent manner (Figure 4E,G). Although these results suggest that the transition from an immortalised to a tumour phenotype is linked to dysregulation of the hsa-miR-620/PML axis, it is necessary to perform functional assays to more accurately validate this hypothesis.

### 3.5. Hsa-miR-620 Originated from the SncmtRNAs Processing

**Figure 5:**
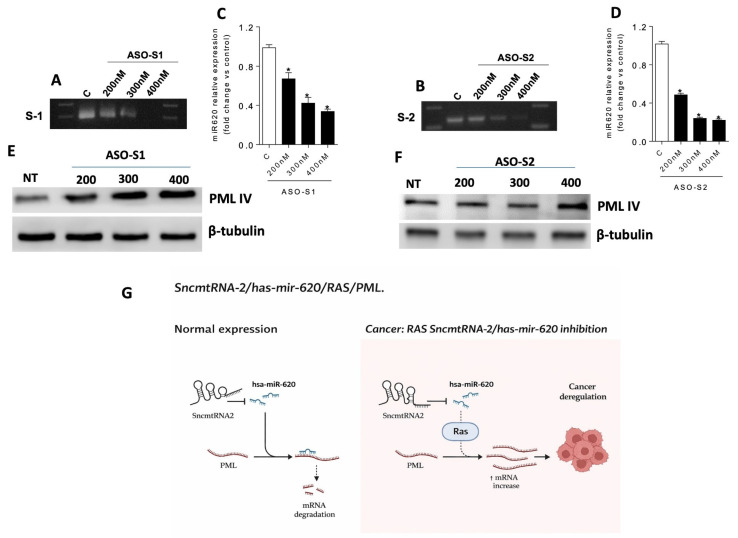
PML is targeted by hsa-miR-620, which is produced by SncmtRNA-2. Ras-18Nco cells were incubated with increasing concentrations (200–400 nM) of ASO-S1 and ASO-S2. (**A**) RT-PCR of SncmtRNA-1 in RAS-18Nco cells treated with increasing concentrations of ASO-S1. (**B**) RT-PCR of SncmtRNA-2 in 18Nco cells treated with increasing concentrations of ASO-S2. (**C**) RT-qPCR amplification of hsa-miR-620 in RAS-18Nco cells treated with increasing concentrations of ASO-S1. (**D**) RT-qPCR amplification of hsa-miR-620 in RAS-18Nco cells treated with increasing concentrations of ASO-S2. (**E**) Expression of PML-IV was analysed by Western blot of total protein extract from RAS-18Nco cells treated with mimic-miR620 at increasing concentrations of ASO-S1. (**F**) Expression of PML-IV was analysed by Western blot of total protein extract from RAS-18Nco cells treated with mimic-miR620 at increasing concentrations of ASO-S2. (**G**) Keratinocytes immortalised by the HPV present high levels of SncmtRNA-2 expression, which is processed to produce an increase in hsa-miR-620, maintaining constant levels of PML-IV expression. However, when these cells are transformed under RAS regulation, SncmtRNA-2 and hsa-miR-620 expression decrease, leading to increased PML-IV expression, which promotes cellular transformation. This axis (SncmtRNA-2, hsa-miR-620/RAS/PML-IV) represents a new pathway of HPV-associated tumourigenesis. The arrow indicates increasing mRNA levels. * Indicates *p* < 0.01.

Different miRNAs originated from the processing of lncRNAs; for example, H19 is a precursor of hsa-miR-675, and Linc-MD1 is a precursor of hsa-miR-206 and hsa-miR-133b [17,18]. To assess whether hsa-miR-620 could originate from S2 processing, we analysed the expression of hsa-miR-620 in 18Nco cells incubated with oligonucleotides (ASO) targeting the S2 molecule (Figure 5). After 48 h of incubation of cells with increasing concentrations of ASO-S2 (200–400 nM), we amplified the S2 transcript by RT-PCR. ASO-S2 treatment at 200 nM induced more than a tenfold decrease in S2 expression (Figure 5B). Correlated with the silencing of S2, hsa-miR-620 expression decreased within the evaluated concentration range (Figure 5D). The silencing of S1 (ASO-S1) also induced downregulation of hsa-miR-620 almost at the same level as ASO-S2 (Figure 5A,C). Finally, the silencing of S2 or S1 leads to an increase in PML-IV levels (Figure 5E,F). Therefore, all results suggest an interplay between S2, hsa-miR-620, and PML-IV that depends on cell status; this axis is altered, as shown in Figure 5G.

## 4. Discussion

Although the role of non-coding mitochondrial RNAs has been studied in various cellular processes, their involvement in cellular immortalisation, as well as their association with the expression of different viral or cellular oncoproteins in inducing this process, has not been fully addressed. Numerous viral proteins from different DNA and RNA viruses, such as HPV, HBV, HCV, and HTLV-1, have been described as playing a key role in activating molecular pathways that drive the transition from a normal to an immortal state, ultimately leading to tumour development [19,20,21].

Current experimental evidence links S1 expression to the proliferative status, while ASncmtRNAs may have a tumour-suppressor-like function [12]. S2 expression is strongly associated with the immortalised cell state. However, the specific role of S2 in immortalisation remains to be elucidated. To investigate this, we used HFK698 and 18Nco cell lines (immortalised by HPV-16 and HPV-18, respectively). Although these immortalised cells escape replicative senescence, they do not form colonies in soft agar or induce tumours in nude mice [17], indicating that they are not yet transformed.

We aimed to induce transformation in both immortalised cell lines by overexpressing oncogenic isoforms of the Ras protein GTPases (H-, K-, and V-Ras) using retroviral vectors. Ras belongs to a family of proteins involved in cell proliferation, differentiation, and survival [18]. Human cells express three Ras genes-H-, K-, and V-RAS—and mutations in these genes are widely described in human cancers [18]. Such mutations result in permanent activation of Ras, leading to deregulated cell proliferation. Several studies have demonstrated that ectopic expression of Ras induces transformation of immortalised cells [22]. Our results indicate that cells overexpressing oncogenic Ras isoforms exhibit altered proliferation rates, enhanced migration capacity, and anchorage-independent growth (Figure 3). The transformed phenotype was significantly more evident in 18Nco immortalised cells overexpressing H-Ras. H-Ras has been described as the isoform most strongly associated with migration and tumourigenesis [23]. 18Nco-RAS cells grew independently of substrate, showed higher DNA synthesis rates, and exhibited increased migratory ability-features consistent with cells acquiring tumoural properties (Figure 3).

Analysis of SncmtRNA expression in these cells revealed that overexpression of H-, K-, or V-Ras resulted in S2 downregulation, consistent with the pattern observed in HeLa cells (Figure 3D vs. Figure 3E). In contrast, expression of Ras isoforms in HFK698 cells slightly altered DNA synthesis rates and migration but did not enable growth independent of anchorage. This suggests that overexpression of Ras isoforms in HFK698 cells is insufficient for full transformation. In these cells, S2 expression remained stable, in contrast to SiHa cells (Figure 3). Our results indicate that S2 expression is associated with the immortalised cell state and is lost as cells progress to transformation. Several studies have described downregulation of long non-coding RNAs (lncRNAs) implicated in cell transformation [24]. In this context, lncRNAs such as GAS5, MEG3, CCND1, MORT, and LincRNA-p21 are downregulated in breast, lung, prostate, and renal cancers, as well as in leukaemia and lymphomas [24]. Previously, we reported that E6 and E7 proteins from high-risk HPV are involved in inducing S-2 overexpression in immortalised cells [7].

Both HPV oncoproteins promote the immortalisation of keratinocytes and fibroblasts in vitro [7,25,26]. Since Ras overexpression induces transformation independently of viral oncoprotein modulation [15], our results suggest that E6/E7 initially trigger SncmtRNA-2 upregulation to promote immortalisation, while a subsequent regulatory mechanism suppresses this transcript during the transition to the transformed state. The selective downregulation of SncmtRNA-2 in 18Nco-RAS cells (Figure 2C) but not HFK698-RAS cells support this model. Identifying the factors and pathways regulating SncmtRNA-2 expression requires further investigation.

Considering that HPV-16 is more prevalent than HPV-18 (50% versus 15%, IARC), our results unexpectedly indicate that HPV-18-immortalised cells are more permissive to Ras-induced transformation.

Previous research has shown that HPV-18 is more oncogenic than HPV-16 [27,28]. Cells transformed with HPV-18 acquire the ability to form colonies in soft agar, a property not observed when using the HPV-16 genome. Comparative studies of the oncoprotein sequences and spatial structures of HPV E6 and E7 have revealed significant differences between them. HPV-18 E6/E7 exhibit a greater capacity to block the functions of p53 and pRB than HPV-16 E6/E7 [25]. In 2010, Teisser et al. demonstrated that E2F5, a known cell-cycle regulator, promotes S-phase entry in the presence of HPV-18 E6/E7 [29]. Within the context of HPV-18 infection, E2F5 enhances genomic instability and cellular malignancy, whereas in HPV-16-infected cells, E2F5 induces G0 arrest [29]. Overexpression of H-Ras has been shown to activate E2F5 [30]. In cells immortalised by HPV-18 and overexpressing oncogenic RAS mutants, E2F5 activity may also increase, potentially contributing to genomic instability and partially explaining the biological basis for the results observed in HPV-18-positive cells. Supporting evidence includes the expression profiles of cytokeratins 14 and 17, as well as the loss of SncmtRNA-2 expression during 18Nco cell transformation.

Previous reports have demonstrated that several oncoviruses, such as Epstein–Barr virus, Herpes Simplex Virus type I, HCV, and KSHV, induce deregulation of numerous miRNAs and lncRNAs involved in antiviral defense, replication, and viral pathogenicity [31]. HBx from HBV upregulates HULC, a cellular lncRNA overexpressed in hepatocellular carcinoma [32]. HPV also modulates cellular miRNAs that regulate its replicative cycle and carcinogenesis. For instance, E6/E7 downregulate hsa-miR-143 and hsa-miR-145 in HPV-induced preneoplastic lesions, suggesting that this downregulation is an early step in cancer development and could serve as an early diagnostic marker [33]. Likewise, hsa-miR-100 levels are reduced in cervical cancer precursor lesions linked to HPV infection [34]. Interestingly, changes in hsa-miR-100 expression are not attributable to E6/E7 oncoproteins, suggesting that other viral proteins may influence this miRNA’s expression [35].

Our results suggest that S2 likely originates from S1 processing, probably in the cytosol. Previously, we reported that ASncmtRNAs interact with Dicer, Drosha, and Argonaute proteins, leading to the formation of mitochondrial-derived miRNAs (mi-to-miRNAs) [36,37]. Therefore, we hypothesised that Dicer and Drosha interact with S1 in the cytosol, while Argonaute facilitates nuclear import, enabling RNA interaction with miRNA pathway proteins to produce S2. The direct interaction of S1 with Argonaute, Dicer, and Drosha warrants further investigation.

Why is it important for transformed cells to downregulate S2 expression? The answer may lie in the potential generation of miRNAs capable of interfering with various cellular pathways. The double-stranded region of SncmtRNAs shares high nucleotide similarity with hsa-miR-620, suggesting that it could serve as a precursor for this miRNA (Figure 4A). Hsa-miR-620 is a poorly characterised miRNA implicated in tumourigenesis of lung and colorectal cancers [38]. Silencing of S1 and S2 transcripts results in downregulation of hsa-miR-620; although both could potentially generate hsa-miR-620, the effect of S1 knockdown might be indirect, as S1 also serves as the precursor of S2. Although dose–response curves and scrambled controls were used, “off-target effects of antisense oligonucleotides and miRNA mimics/antagomirs cannot be fully ruled out, and future work will incorporate additional sequence-distinct ASOs and rescue experiments to further refine the specificity of the SncmtRNA-2/miR-620/PML axis. 18Nco-RAS cells exhibit downregulation of hsa-miR-620 alongside S2 (Figure 3), whereas HFK698-RAS cells maintain expression of both hsa-miR-620 and S2 (Figure 3).

Generation of miRNAs from lncRNAs has been well documented. He et al. (2008) reported 22 lncRNAs encoding miRNAs in mice [39], and Uva et al. (2013) identified a lncRNA named *Bic* that is processed to generate rat miR-155 [40]. Subsequent studies have identified other human lncRNAs that produce miRNAs, including *Linc-MD1* (miR-206 and miR-133b) [41], *H19* (miR-675), and *MRP* (RMRP-S1 and RMRP-S2) [42]. Our work explores the potential of S2 as a precursor of hsa-miR-620, and we have also identified other putative miRNA candidates within S2 that merit further study. In silico analysis suggests that PML is a target of hsa-miR-620 (Figure 4A). This finding is supported by our in vitro assays, where 18Nco-RAS cells show upregulation of PML along with downregulation of S2 and hsa-miR-620 (Figure 4). HFK698-RAS cells, which did not fully transition to a transformed state, maintained expression of PML, S2, and hsa-miR-620 (Figure 4). To test direct binding of miR-620 to PML, luciferase reporter assays using both wild-type and mutant PML 3′UTR constructs will be required.

The expression of S2 in HPV-immortalised cells likely results in high levels of hsa-miR-620, maintaining low levels of PML-IV and thereby preserving the immortalised state. PML is a multifunctional protein described as a tumour suppressor with antiviral properties. Interestingly, HPV proteins E6, E7, and L2 interact with PML-IV and induce its relocalisation within nuclear bodies [16]. This raises an important question: why would an oncovirus need to interact with an antiviral protein to replicate? Wimmer et al. showed that PML-IV participates in cell transformation induced by adenovirus infection [43]. These results suggest that PML may have a dual role, acting more like an oncoprotein than a tumour suppressor, at least in DNA virus–mediated cell transformation.

In summary, our results support a new model in which immortalised cells may retain their immortalised status to prevent full transformation. Future studies will be required to perform PML knockdown in Ras-transformed cells and PML-IV overexpression in immortalised cells to determine whether PML-IV is necessary and/or sufficient for the phenotypes described. Future research should assess whether non-viral immortalised cells also utilise S2 overexpression to maintain their immortalised state, or whether this pathway is uniquely exploited by viruses to control the host cell.

## 5. Conclusions

Our findings support a model in which Ras-induced transformation of HPV-immortalised cells is associated with downregulation of the SncmtRNA-2/hsa-miR-620 axis and increased PML-IV expression, indicating that this ncRNA/miRNA–PML axis may constitute a plausible regulatory pathway contributing to the transformation process, rather than providing evidence that PML-IV functions as a necessary effector. Overexpression of Ras oncogenes in these cells leads to reduced SncmtRNA-2 and miR-620 levels, accompanied by increased PML-IV expression, indicating a critical regulatory switch. This study reveals a novel role for PML-IV as a candidate downstream effector of Ras-mediated transformation in HPV-18-immortalised cells. The upregulation of PML-IV resulting from suppression of the SncmtRNA-2/miR-620 axis underscores the complex interplay between cellular and viral factors in promoting oncogenesis.

Importantly, our data suggest that the ability of Ras overexpression to induce transformation depends on the specific HPV background. HPV-18-immortalised cells were more susceptible to transformation than HPV-16-immortalised cells, highlighting the involvement of additional cellular and viral components that warrant further investigation.

These findings broaden our understanding of the contribution of non-coding RNAs to viral oncogenesis and emphasise the importance of mitochondrial-derived transcripts in regulating cellular transformation. Future research should focus on elucidating the precise molecular mechanisms through which SncmtRNA-2 and miR-620 modulate PML-IV expression and activity.

Although the role of Ras in cellular transformation has been extensively characterised, the mechanism or mediator by which Ras overexpression results in down-regulation of SncmtRNA-2 remains unknown. Future studies should address the mechanism underlying Ras-mediated negative regulation of SncmtRNA-2 expression.

This study has several limitations that should be considered when interpreting the findings. Direct binding of miR-620 to the PML 3′UTR was not assessed, and therefore the regulatory relationship between these molecules remains unconfirmed. Likewise, the necessity or sufficiency of PML for Ras-induced transformation was not directly evaluated, leaving its functional contribution unresolved. In addition, oligonucleotide-based approaches were performed at relatively high concentrations, and the use of additional antisense oligonucleotides, along with formal rescue experiments, would strengthen the robustness of future mechanistic analyses.

## Data Availability

The data presented in this study are available on request from the corresponding author. The data are not publicly available due to restrictions regarding the privacy of the human participants and ethical reasons.

## Supplementary Materials

The following supporting information can be downloaded at: https://www.mdpi.com/article/10.3390/medicina62010110/s1, Figure S1: Overexpression of Ras in HPV-immortalised cells. HPV-16/18 immortalised cells were transduced with a lentivirus encoding GFP (ZsGreen) and H, K, and V isoforms of RAS. (A) Flow cytometry analysis. Analysis of transduction efficiency after sorting of 18Nco-RAS, HFK698-RAS, and HFK-HRAS cells. (B) Western Blot of 18Nco cells expressing H, K, and V-RAS. (D) Western Blot of HFK698 cells expressing H, K, and Vras. (F) Western Blot of HFK cells expressing H-RAS. (C, E and G) The graphs show Ras expression relative to β-actin. The relative expression of RAS was calculated from three independent assays. C (control) in graph correspond to WT (wild type cells) in Blots. * Indicates p<0.01. Figure S2: Expression of cytokeratin 14/17 in HPV-immortalised and transformed cells. Immunofluorescence microscopy showing localization of cytokeratin 14 (CK-14) and 17 (CK-17) in HPV-immortalised and RAS-transformed cells. Antibodies used for staining are described in the Materials and Methods section. Scale bar represents 20 μm.
