# Supplementary material for: HPV-18-Immortalised Cells Require the Downregulation of the SncmtRNA-2/Hsa-miR-620 Axis During Cell Transformation
Source: Medicina (Kaunas). 2026 Jan 4;62(1):110. doi: 10.3390/medicina62010110 (PMC12843629; doi:10.3390/medicina62010110)
Supplement: Supplementary file 1 [file medicina-62-00110-s001.zip › medicina-3948528-supplementary.pdf]

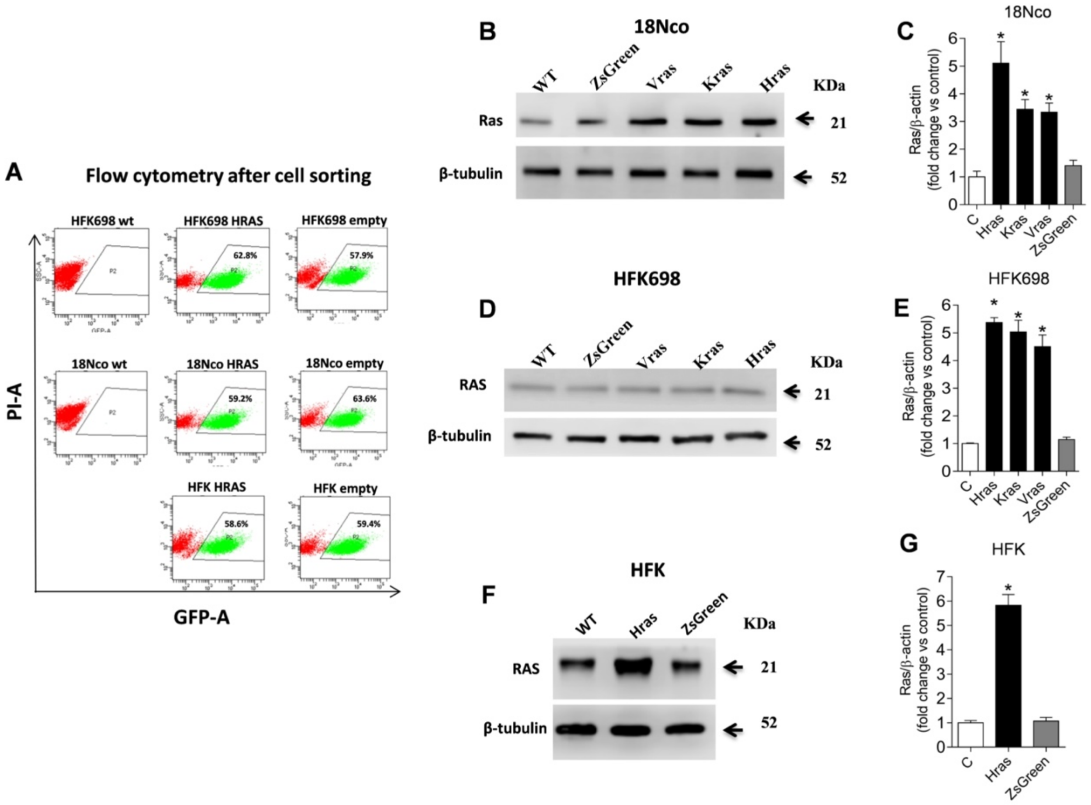

Supplementary figure S 1.- ***Overexpression of Ras in HPV-immortalized cells.*** *HPV-16/18 immortalized cells were transduced with a lentivirus coding GFP (ZsGreen) and H, K and V-isoforms of RAS. A) FALTA COMPLETAR ESTO. B) Western Blot of 18Nco cells expressing H, K and Vras. D) Western Blot of HFK698 cells expressing H, K and Vras. F) Western Blot of HFK cells expressing H-RAS. C, E and G) The graphs show the expression of Ras relative to B-actin. The relative expression of Ras was calculated from three independent assays. * Indicates p<0.01.*

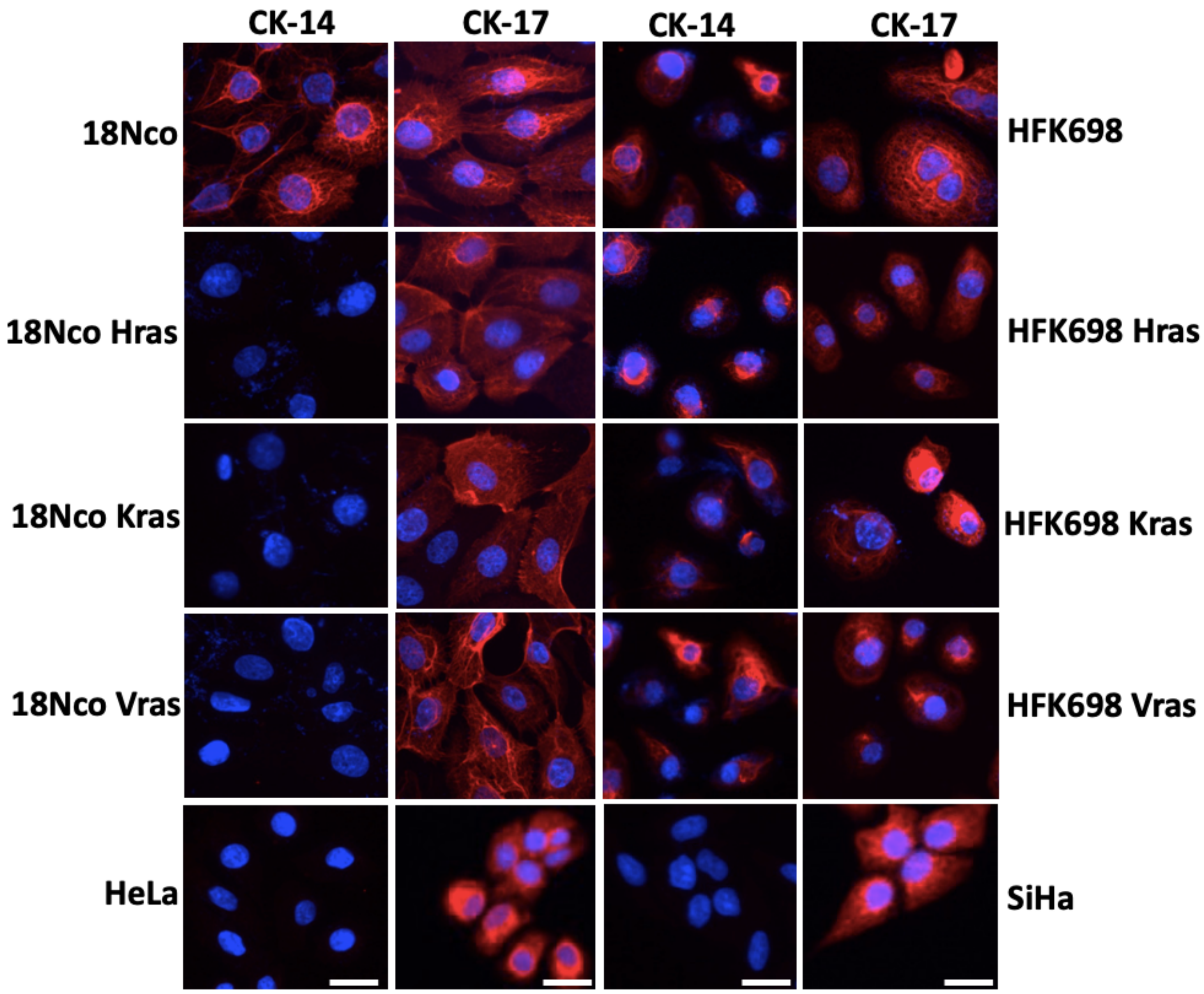

Supplementary figure S2. Expression of cytokeratin 14/17 in HPV immortalized and transformed cells*.* *Immunofluorescence microscopy showing localization of cytokeratin 14 (CK-14) and 17 (CK-17) in HPV-immortalized and RAS-transformed cells. Antibodies used for staining are described in materials and methods. Scale bar represents 20 µm*
